# No difference in COVID-19 treatment outcomes among current methamphetamine, cannabis and alcohol users

**DOI:** 10.1186/s42238-023-00193-w

**Published:** 2023-06-19

**Authors:** Ann Rydberg, Christopher A. Dodoo, Terry D. Schneekloth, Osama A. Abulseoud

**Affiliations:** 1 Alix School of Medicine at Mayo Clinic, Phoenix, AZ 58054 USA; 2 Quantitative Health Sciences, Mayo Clinic Arizona, Phoenix, AZ 58054 USA; 3 Department of Psychiatry and Psychology, Mayo Clinic Arizona, 5777 E Mayo Blvd, Phoenix, AZ 58054 USA; 4 Department of Neuroscience, Mayo Clinic Graduate School of Biomedical Sciences, CRB, Scottsdale, AZ 85259 USA

**Keywords:** COVID-19, Methamphetamine, Cannabis, Delirium, Vaccination, Length of stay, Alcohol

## Abstract

**Background:**

Poor outcomes of COVID-19 have been reported in older males with medical comorbidities including substance use disorder. However, it is unknown whether there is a difference in COVID-19 treatment outcomes between patients who are current cannabis users, excessive alcohol drinkers and those who use a known hazardous stimulant such as methamphetamine (METH).

**Methods:**

Electronic medical records (EMR) of COVID-19 patients with current METH (*n* = 32), cannabis (*n* = 46), and heavy alcohol use (*n* = 44) were reviewed. COVID-19 infection was confirmed by positive SARS-CoV-2 PCR test, current drug use was confirmed by positive urine drug testing, and alcohol use was identified by a blood alcohol concentration greater than 11 mg/dl.

Multivariate linear regression models as well as the firth logistic regression models were used to examine the effect of substance use group (METH, cannabis, or alcohol) on treatment outcome measures.

**Results:**

A total of 122 patients were included in this analysis. There were no significant differences found between drug groups in regards to key SARS-CoV-2 outcomes of interest including ICU admission, length of stay, interval between SARS-CoV-2 positive test and hospital discharge, delirium, intubation and mortality after adjusting for covariates. About one-fifth (21.9% in METH users, 15.2% in cannabis users, and 20.5% in alcohol users) of all patients required ICU admission. As many as 37.5% of METH users, 23.9% of cannabis users, and 29.5% of alcohol users developed delirium (*P* = 0.4). There were no significant differences between drug groups in COVID-19 specific medication requirements. Eight patients in total died within 10 months of positive SARS-CoV-2 PCR test. Two patients from the METH group (6.3%), two patients from the cannabis group (4.3%), and four patients from the alcohol group (9.1%) died.

**Discussion:**

The study outcomes may have been affected by several limitations. These included the methodology of its retrospective design, relatively small sample size, and the absence of a COVID-19 negative control group. In addition, there was no quantification of substance use and many covariates relied on clinical documentation or patient self-report. Finally, it was difficult to control for all potential confounders particularly given the small sample size.

**Conclusion:**

Despite these limitations, our results show that current METH, cannabis, and heavy alcohol users in this study have similar treatment outcomes and suffer from high morbidity including in-hospital delirium and high mortality rates within the first-year post COVID-19. The extent to which co-morbid tobacco smoking contributed to the negative outcomes in METH, cannabis, and alcohol users remains to be investigated.

**Supplementary Information:**

The online version contains supplementary material available at 10.1186/s42238-023-00193-w.

## Introduction

The Coronavirus disease 2019 (COVID-19) pandemic caused by Severe Acute Respiratory Syndrome Corona Virus (SARS CoV-2) began at a unique time when the healthcare field was actively struggling with the substance use pandemic (Volkow 2020; Berlin et al. 2020). Patients suffering from a substance use disorder (SUD) are at significantly higher risk for developing adverse effects of COVID-19 including more severe illness and higher mortality rates compared to patients without SUD (Baillargeon et al. 2021; Benzano et al. 2021; Catalan et al. 2022; Wang et al. 2021; Parohan et al. 2020; Wolff et al. 2021; Zhou et al. 2021). Nonetheless, most countries (Balhara et al. 2020) have considered alcohol and tobacco sales during COVID-19 lockdown as essential products (Stockwell et al. 2020). Wang et al. (Wang et al. 2021) reported that individuals with recent SUD diagnosis (made in the last year) had significantly higher risk of developing COVID-19 compared to patients without recent SUD diagnosis, after adjusting for age, gender, race, and insurance types. The highest risk was observed in patients with opioid use disorder followed by tobacco, then alcohol, cocaine, and cannabis use disorders. Furthermore, among those patients with COVID-19 and recent SUD diagnosis, 43.8% required hospitalization and 9.5% died of COVID-19 (Wang et al. 2021). This severe course of illness and poor outcome has been reported in patients with SUD in general (Baillargeon et al. 2021) and in nicotine smokers specifically (Benzano et al. 2021; Karanasos et al. 2020). Two systematic reviews documented that mental disorders are associated with increased COVID-19 mortality.

Fond et al. reviewed 16 studies (*n* = 19,086 patients), COVID-19 mortality was increased among patients with mental health disorders compared with patients without mental health disorders [odds ratio = 1.75, 95% CI, (1.40–2.20), *P* < 0.05)] (Fond et al. 2021). Vai et al. reviewed 23 studies comprising 1,469,731 COVID-19 patients including 43,938 COVID-19 patients with mental illness. The authors reported that the presence of any mental disorder was associated with an increased risk of COVID-19 mortality [odds ratio = 2·00, 95% CI (1·58–2·54); I^2^ = 92·66%)] (Vai et al. 2021).

However, it is not yet known whether current users of a commonly abused substance such as cannabis, have different COVID-19 courses or treatment outcomes especially when compared to users of a hard drug such as methamphetamine (METH) or to heavy alcohol users. The users of these two psychogenic substances: METH and cannabis, tend to differ in many aspects. Differences in COVID-19 risk factors between these two populations include male sex (Brecht et al. 2004) and high prevalence of hepatitis C (Gonzales et al. 2006) in METH users and possible obesity among cannabis users (Fearby et al. 2022). In fact, current use of either cannabis or METH is associated with conditions known to be associated with poorer COVID-19 outcomes such as tobacco smoking (Yen and Chong 2006; Badiani et al. 2015) and pulmonary conditions. Substance-specific risk factors include hesitancy to receive COVID-19 vaccine among cannabis users (Wolff et al. 2021; Schauer et al. 2021; Stack et al. 2021; Bolinski et al. 2022), and altered immune response to infection among METH users, rendering them subject to more severe and prolonged illness (Courtney and Ray 2014; Kaye et al. 2007; Kolaitis et al. 2021; Prakash et al. 2017). In contrast, a recent study reported that current cannabis users hospitalized with COVID-19 (*n* = 69) had better clinical outcomes compared with non-users, including a decreased need for ICU admission and mechanical ventilation (Shover et al. 2022).

In this retrospective study, we aimed to compare treatment outcome differences between three cohorts of COVID-19 patients who received care at Mayo Health System between April 2020 and May 2022 and who had positive drug screen for either cannabis, METH, or ethanol. We hypothesized that the use of any of the three substances of interest: METH, cannabis, or alcohol could result in complicated COVID-19 treatment course, although we speculated that adverse outcomes would be more prevalent in METH users than cannabis or alcohol users.

## Methods

This study was approved by the Institutional Review Board of the Mayo Clinic and COVID-19 Research Task Force (ID: 21–010940). Electronic medical records (EMR) of adult COVID-19 patients with current METH or cannabis use who received care at Mayo Clinic between April 2020 and September 2021 were reviewed. We surveyed additional patients with detected high blood alcohol concentrations at the time of COVID-19 infection in a hospital setting. This cohort was identified as being in the hospital at the time of COVID-19 infection, so while the hospitalization rate for COVID-19 is 100% the other metrics are comparable to the METH and cannabis users assessed. Patients were included if they had a positive toxicology drug screen and positive SARS-CoV-2 PCR test with available electronic medical record (EMR) documentation of care at the time of positive COVID-19 infection. Patients were identified as being current users of the substances of interest (alcohol, METH, or cannabis) through a blood alcohol concentration greater than the minimum detection level of 11 mg/dL or positive urine drug screen without prescription medication (such as amphetamines for attention deficit hyperactivity disorders) that confound urine drug testing. If a patient was prescribed such medications, confirmatory drug testing was required to include the patient in the methamphetamine cohort. Patients who were found to test positive for multiple substance on urine drug screen and/or blood alcohol concentration were considered as duplicate cases and were eliminated from review. The first identified encounter for each patient was included in the analysis if there were multiple encounters for a single patient. The following data were extracted from EMR: demographic characteristics, presenting complaints including COVID-19 symptoms, substance intoxication, pre-existing medical or psychiatric conditions, COVID-19 course including hospitalization, ICU admission, delirium or altered mental status or significant agitation necessitating anti-psychotics, COVID-19 pharmacological management, and treatment outcomes. This information was readily available in the patient’s discharge summary which included relevant testing results, medications required, follow-up recommendations, documentation of inpatient delirium, oxygen requirements, and ICU status.

Information about comorbid medical and psychiatric conditions including substance use was collected via the patients’ discharge summaries with updated hospital problem lists. If a patient had a medical or psychiatric condition noted in a separate encounter this was not included as we strove to include only active problem (often chronic medical conditions) noted at the time of COVID-19 infection. Comorbid conditions were then classified into organ-system or psychiatric disorder-based categories. Current or former tobacco use was identified by self-report as patients are asked about smoking status when admitted to the hospital or evaluated in clinic. Patients who were currently or previously smoking tobacco-based products (i.e., cigarettes, cigars) or using chew tobacco were included. Pain disorders were identified when a patient had a chronic pain related diagnosis on their hospital problem list at the time of discharge. These conditions included fibromyalgia, chronic pain syndrome, chronic abdominal pain, chronic back pain, etc. Medical conditions that did not fit into a clear organ system category were organized into an “other category.” These conditions included vitamin deficiency, fragility, dehydration, skin ulcer, organ transplant, history of falling, developmental delay, visual issues, sarcoidosis, extensive surgical history, and other assorted conditions.

Substance use was documented if it was included in a patient’s problem list. Substance use was subsequently classified into stimulant use including methamphetamine, cocaine, and unspecified stimulant use; alcohol use; and use of sedatives or hypnotic medications including opiates, benzodiazepines, and barbiturates. Psychiatric conditions that did not fit into defined categories were placed into an “other” category. These included adjustment disorder, Attention-Deficit Disorder/Attention-Deficit Hyperactivity Disorder (ADD/ADHD), dissociative disorder, sleep disorders, insomnia, ineffective coping, eating disorder, history of physical or psychological abuse, gender dysphoria, autism spectrum disorder, and fetal alcohol syndrome. Data was also collected for psychiatric medications that a patient was prescribed on date of discharge. Antidepressant medications were further subdivided into Selective Serotonin Reuptake Inhibitor (SSRI) and non-SSRI medications. Non-SSRI antidepressant medications used in the two patient cohorts consisted of mirtazapine, duloxetine, bupropion, and amitriptyline. Non-benzodiazepine anxiolytics included venlafaxine, desvenlafaxine, hydroxyzine, and buspirone. ADD medications included stimulants such as amphetamine/dextroamphetamine salts and non-stimulant medication such as guanfacine. Sleep medications included trazodone, zolpidem, and diphenhydramine when used for sleep only. Post-Traumatic Stress Disorder (PTSD) specific medications was only prazosin in our cohorts. Other psychiatric medications consisted of antiepileptics, Parkinson’s medications, or pain medications used for primary psychiatric conditions.

If a patient was not admitted to the hospital at the time of COVID-19 infection, a problem list and current medications were taken from the closest visit with complete documentation within a maximum of 7 months. If a patient later developed a medical or psychiatric condition, it was not included in our analysis as it was not documented during the acute COVID-19 infection. EMR documentation was considered complete if there was complete documentation of hospitalization during COVID-19 infection, positive drug test confirmed with further testing after initial urine drug screen (UDS), and a complete record of active medical and psychiatric problems. If EMR documentation was not complete, the patient was excluded from the analysis.

### Statistical analysis

Continuous variables were summarized using means, standard deviation, median, inter-quartile range (IQR) and range. For categorical variables, frequencies and percentages were used. To assess the bivariate association between each of the categorical variables and the three study groups, a chi – squared test was used; the one-way ANOVA was used to assess the differences in means of each of the normally distributed continuous variables across the three study groups. For non–normally distributed continuous variables, the Kruskal–Wallis test was preferred.

Covariates of interest extracted were demographics (Age, BMI, Sex, Race, Ethnicity, Marital status, employment status, tobacco use, received vaccination before and after hospitalization), patients’ medications, and comorbid medical and psychiatric conditions. Only covariates that were statistically significant (Age, Sex, Vaccination before hospital admission, tobacco use, psych symptoms AMS, alcohol related disorders, stimulant usage, benzodiazepines s given) were eligible to be included in the multivariable model.

Multivariate analysis models were used to examine the effect of substance use group (METH, cannabis or alcohol) on treatment outcome measures: ICU length of stay, interval between SARS-CoV-2 positive test and hospital discharge, ICU admission, delirium, intubation and mortality. For ICU admissions, delirium, intubation, and mortality, firth Logistic regression model was used. Firth’s logistic regression reduces the bias in maximum likelihood estimates of the coefficients usually occurring when there is a strong imbalance in the outcome, as was in the case of mortality, delirium, intubation and ICU admission. (Puhr et al. 2017) Estimates from the model were reported as odds ratios (OR) along with their 95% confidence interval (CI).

The outcomes of ICU LOS and Interval between SARS-CoV-2 positive test and discharge were log transformed before performing a multivariable analysis. The multiple linear regression model was used to assess the association between ICU LOS and the interval between SARS-CoV-2 positive test and discharge. Estimates from these models were reported as regression coefficients (RC) along with their 95% CI.

*P* values ≤ 0.05 were considered statistically significant for this study and all analyses were performed using SAS V 9.4 from SAS Institute Inc 2013, Cary, NC.

## Results

### Demographics

A total of 182 cases from 122 patients were identified for this study. Of these, 60 were considered duplicate cases. After eliminating duplicates, there were a total of 122 (METH: *n* = 32, 26.2%, cannabis: *n* = 46, 37.7%, and alcohol: *n* = 44, 36.1%) patients used in this analysis.

All patient groups consisted predominantly of middle aged [METH vs. cannabis vs. alcohol: 40.5 ± 12.0 vs. 41.2 ± 17.1 vs. 44.8 ± 12.5 years respectively], non-Hispanic Caucasians with significantly more female cannabis users, 47.8%, than METH or alcohol users (15.6% and 22.7% respectively, *P* = 0.004). Most METH, cannabis, and alcohol users were single, unemployed, or disabled. METH users had a mean BMI of 26.9 ± 6.15 kg/m^2^, cannabis users had a mean BMI of 29.4 ± 7.76 kg/m^2^, and alcohol users had a mean BMI of 27.6 ± 6.97 kg/m^2^. However, obesity (BMI between 30.1–40 kg/m^2^) was present in 12.9% of the METH users, 26.1% of cannabis users, and 23.8% of alcohol users and morbid obesity (BMI > 40 kg/m^2^) was present in 6.5% of METH, 13.0% of cannabis, and 7.1% of alcohol users. There was no statistically significant difference in BMI among the three groups, *P* = 0.3. Most current METH, cannabis, and alcohol users with COVID-19 infection reported current or past tobacco smoking (78.1%, 80.4%, and 75.0% respectively, *p* = 0.8). Significantly more current alcohol users (38.6%) reported receiving COVID-19 vaccination prior to hospital admission compared to current cannabis (8.7%) and METH users (6.3%), *p* = 0.0001 (Table 1). There was no significant difference among groups in COVID-19 vaccination rates following hospital visit (*p* = 0.3),

**Table 1:** Sociodemographic and clinical characteristics of study patients

	METH (*N* = 32) n (%) or mean (SD)	Cannabis (*N* = 46) n (SD or %)	Alcohol (*N* = 44) n (SD or %)	Total (*N* = 122) n (SD or %)	*P*-value
**Age at COVID test (in years)**					0.3481^1^
Mean (SD)	40.5 (11.98)	41.2 (17.06)	44.8 (12.45)	42.3 (14.27)	
Range	20.5–59.1	18.0- 74.6	21.0–77.0	18.0–77.0	
**Age groups**					0.0553^2^
18–30 years	7 (21.9%)	16 (34.8%)	4 (9.1%)	27 (22.1%)	
30.1-40 years	10 (31.3%)	9 (19.6%)	13 (29.5%)	32 (26.2%)	
> 40 years	15 (46.9%)	21 (45.7%)	27 (61.4%)	63 (51.6%)	
**Sex**					0.0038^2^
Male	27 (84.4%)	24 (52.2%)	34 (77.3%)	85 (69.7%)	
Female	5 (15.6%)	22 (47.8%)	10 (22.7%)	37 (30.3%)	
**Race**					0.4146^2^
White	28 (87.5%)	36 (78.3%)	32 (72.7%)	96 (78.7%)	
African American	1 (3.1%)	7 (15.2%)	5 (11.4%)	13 (10.7%)	
Other	3 (9.4%)	3 (6.5%)	6 (13.6%)	12 (9.8%)	
Unknown/ Missing	0 (0.0%)	0 (0.0%)	1 (2.3%)	1 (0.8%)	
**Ethnicity**					0.4313^2^
Hispanic	1 (3.1%)	7 (15.2%)	3 (6.8%)	11 (9.0%)	
Not Hispanic or Latino	30 (93.8%)	38 (82.6%)	40 (90.9%)	108 (88.5%)	
Unknown/ Missing	1 (3.1%)	1 (2.2%)	1 (2.3%)	3 (2.5%)	
**Marital Status**					0.0895^*2*^
Single	22 (68.8%)	23 (50.0%)	17 (38.6%)	62 (50.8%)	
Married/ Life Partnership	4 (12.5%)	14 (30.4%)	14 (31.8%)	32 (26.2%)	
Divorced	4 (12.5%)	5 (10.9%)	9 (20.5%)	18 (14.8%)	
Other	0 (0.0%)	3 (6.5%)	4 (9.1%)	7 (5.7%)	
Unknown/ Missing	2 (6.3%)	1 (2.2%)	0 (0.0%)	3 (2.5%)	
**Employment Status**					0.0043^2^
Employed	10 (31.3%)	22 (47.8%)	9 (20.5%)	41 (33.6%)	
Not Employed	16 (50.0%)	10 (21.7%)	18 (40.9%)	44 (36.1%)	
Student/ Disabled/ Retired	4 (12.5%)	10 (21.7%)	5 (11.4%)	19 (15.6%)	
Unknown/ Missing	2 (6.3%)	4 (8.7%)	12 (27.3%)	18 (14.8%)	
**BMI (Body Mass Index)** kg/m^2^					0.2558^1^
N (Missing)	31 (1)	46 (0)	42 (2)	119 (3)	
Mean (SD)	26.9 (6.15)	29.4 (7.76)	27.6 (6.97)	28.1 (7.12)	
Range	18.9 -44.3	14.7—46.1	15.3—44.4	14.7 -46.1	
**BMI groups**					0.5262^2^
< 18 kg/m^2^ (Underweight)	0 (0.0%)	2 (4.3%)	2 (4.8%)	4 (3.4%)	
18.0–25.0 kg/m^2^ (Normal Weight)	18 (58.1%)	15 (32.6%)	17 (40.5%)	50 (42.0%)	
25.1–30.0 kg/m^2^ (Overweight)	7 (22.6%)	11 (23.9%)	10 (23.8%)	28 (23.5%)	
30.1–40 kg/m^2^ (Obese)	4 (12.9%)	12 (26.1%)	10 (23.8%)	26 (21.8%)	
> 40 kg/m^2^ (Morbidly Obese)	2 (6.5%)	6 (13.0%)	3 (7.1%)	11 (9.2%)	
Missing	1	0	2	3	
**Tobacco Use (current or past)**	25 (78.1%)	37 (80.4%)	33 (75.0%)	95 (77.9%)	0.8240^2^
Received vaccination before hospital visit	2 (6.3%)	4 (8.7%)	17 (38.6%)	23 (18.9%)	0.0001^2^
Received vaccination after hospital visit (*n* = 99)	12 (40.0%)	25 (59.5%)	13 (48.2%)	50 (50.5%)	0.2527^2^

*SD* Standard deviation

^1^ANOVA F-test *p*-value

^2^Chi-Square *p*-value

### Comorbid medical and psychiatric conditions

There were no significant differences observed in medical comorbidities between the three cohorts aside from in “other,” non-classified medical conditions. These occurred at a rate of 12.5% in METH users, 21.7% in cannabis users, and 25.0% in alcohol users (*P* < 0.0001). All cohorts had high rates of cardiovascular conditions: 43.0% in METH users, 54.3% in cannabis users, and 63.6% in alcohol users (*P* = 0.2) (Supplementary Table 1).

Likewise, all cohorts had high rates of gastrointestinal (GI) conditions (40.6% vs. 30.4% vs. 43.2%, *P* = 0.4). Hepatitis C and liver dysfunction were the most common GI conditions in the METH group, GERD, hepatic dysfunction, and IBD were the most prevalent gastrointestinal disorders in the cannabis group, and the most prevalent gastrointestinal disorders in alcohol users were gastritis, alcoholic steatosis, and non-alcoholic steatosis (Supplementary Table 1).

There were more alcohol users with a history of psychotic disorders compared with METH and cannabis users (31.8% vs. 12.5% and 13.0% respectively, *P* = 0.04). There were also significantly more alcohol users with a documented history of alcohol use disorder or alcohol-related disorders on their discharge problem lists (93.2%) compared with METH users (28.1%) and cannabis users (15.2%) (*P* < 0.0001). Likewise, there were significantly more METH users with a history of stimulant use on their active problem lists (62.5%) compared to cannabis users (17.4%) and alcohol users (11.4%) (*P* < 0.0001). Of note, over half of all groups had diagnosed mood disorders included in their active problem lists. Likewise, around a third of all groups had anxiety disorders. There were no significant differences in the prevalence of mood disorders or anxiety disorders between groups, *P* = 0.9 and 0.4 respectively (Supplementary Table 1).

### Reason for hospital visit

Drive-through COVID testing or COVID related symptoms (e.g., headache, cough, shortness of breath) constituted a frequent reason for hospital visit (25.0% in METH users vs. 37.0% in cannabis users vs. 15.9% in alcohol users), *P* = 0.07. More patients in the alcohol group presented with substance intoxication or withdrawal (56.8% of alcohol users vs. 18.8% of METH users vs. 0% prevalence in the cannabis group, *P* =  < 0.0001). Frequently abused substances causing intoxication as a presenting complaint besides METH were heroin, prescription opiates and alcohol. There were no significant differences in presentations for trauma, injury, infection, diabetic ketoacidosis, nausea or vomiting or dehydration, pain, labor and delivery, seizure disorders, heart failure, pneumothorax, or other complaints between the three patient groups (Table 2).

**Table 2:** Reason for hospital visit

	METH (*N *= 32) n (%)	Cannabis (*N* = 46) n (%)	Alcohol (*N* = 44) n (%)	Total (*N* = 122) n (%)	*P*-value^1^
COVID related symptoms	8 (25.0%)	17 (37.0%)	7 (15.9%)	32 (26.2%)	0.07
Substance intoxication, withdrawal	6 (18.8%)	0 (0.0%)	25 (56.8%)	31 (25.4%)	< .0001
Trauma or injury or infection	1 (3.1%)	1 (2.2%)	2 (4.5%)	4 (3.3%)	0.8
Diabetic ketoacidosis	4 (12.5%)	1 (2.2%)	1 (2.3%)	6 (4.9%)	0.06
Nausea, vomiting or dehydration	2 (6.3%)	6 (13.0%)	3 (6.8%)	11 (9.0%)	0.4
Pain	2 (6.3%)	0 (0.0%)	5 (11.4%)	7 (5.7%)	0.06
Labor & delivery	0 (0.0%)	3 (6.5%)	0 (0.0%)	3 (2.5%)	0.07
Seizure	0 (0.0%)	1 (2.2%)	0 (0.0%)	1 (0.8%)	0.4
Heart Failure or shock	0 (0.0%)	1 (2.2%)	0 (0.0%)	1 (0.8%)	0.4
Pneumothorax	0 (0.0%)	1 (2.2%)	0 (0.0%)	1 (0.8%)	0.4
Other complaints	0 (0.0%)	2 (4.3%)	0 (0.0%)	2 (1.6%)	0.1

^1^Chi-Square *p*-value

### Hospital course and psychiatric medications at time of discharge

Over half of the patients in the METH and cannabis groups were hospitalized (71.9% of METH users vs. 58.7% of cannabis users). All of the alcohol using cohort were identified while hospitalized, so the hospitalization rate for this group was 100%. The other metrics are comparable to the METH and cannabis using cohorts. About a quarter of all groups (25.0% in cannabis users vs. 26.1% in METH users vs. 25.0% in alcohol users) received COVID-specific medications, *P* = 0.99. Cannabis users had the greatest interval between SARS-CoV-2 positive test and hospital discharge with a mean of 9.4 days (SD 9.2) compared to 5.9 days (SD 6.3) and 4.7 days (SD 4.7) in METH and alcohol users respectively (*P* = 0.02) (Supplementary Table 2).

Eight patients in total died within 10 months of positive SARS-CoV-2 PCR test. Two patients from the METH group (6.3%), two patients from the cannabis group (4.3%), and four patients from the alcohol group (9.1%) died. There was no significant difference in mortality among the groups, *P* = 0.7. More METH users required benzodiazepines than their cannabis user counterparts (46.9% vs 26.1%). Alcohol users required the most benzodiazepines of any group through Clinical Institute Withdrawal Assessment of Alcohol Scale, Revised (CIWA-Ar) protocol (68.2%) (*P* = 0.0003) (Sullivan et al. 1989). There were no significant differences in the frequency of antipsychotic (21.9% vs. 28.3% vs. 20.5%) or opiate pain medication (21.9% vs. 34.8% vs. 25.0%) requirements between the three groups (*P*-values = 0.7 and 0.4 respectively). Delirium occurred in 37.5% of the METH users, in 23.9% of the cannabis users, and in 29.5% of the alcohol users (*P* = 0.4) (Supplementary Table 2).

About half of the patients in all groups (53.1% in METH users vs. 65.2% in cannabis users vs. 59.1% in alcohol users, *P* = 0.6) were prescribed a psychiatric medication at time of discharge, most commonly antidepressants (28.1% vs. 45.7% vs. 40.9%, *P* = 0.3) and antipsychotics (18.8% vs. 17.4% vs. 22.7%, *P* = 0.8) There were no significant differences in psychiatric medications at the time of discharge between the three cohorts (Table 3).

**Table 3:** Psychiatric medications being taken at hospital discharge

	Group				
	METH (*N* = 32) n (%)	Cannabis (*N* = 46) n (%)	Alcohol (*N* = 44) n (%)	Total (*N* = 122) n (%)	*P*-value
Any Psychiatric Medications	17 (53.1%)	30 (65.2%)	26 (59.1%)	73 (59.8%)	0.5587^1^
Antidepressants	9 (28.1%)	21 (45.7%)	18 (40.9%)	48 (39.3%)	0.2865^1^
Antipsychotics	6 (18.8%)	8 (17.4%)	10 (22.7%)	24 (19.7%)	0.8071^1^
Mood Stabilizers	3 (9.4%)	1 (2.2%)	1 (2.3%)	5 (4.1%)	0.2151^1^
Anxiolytics	4 (12.5%)	8 (17.4%)	11 (25.0%)	23 (18.9%)	0.3688^1^
Attention deficit disorder medications	2 (6.3%)	1 (2.2%)	3 (6.8%)	6 (4.9%)	0.5483^1^
Sleep Medications	4 (12.5%)	7 (15.2%)	7 (15.9%)	18 (14.8%)	0.9122^1^
Benzodiazepines	3 (9.4%)	4 (8.7%)	3 (6.8%)	10 (8.2%)	0.9115^1^
PTSD (Post-traumatic stress disorder) medications	0 (0.0%)	1 (2.2%)	2 (4.5%)	3 (2.5%)	0.4447^1^
Others (anticonvulsants or Parkinson disease or pain medications used off label)	2 (6.3%)	1 (2.2%)	3 (6.8%)	6 (4.9%)	0.5483^1^

^1^Chi-Square *p*-value

### Effect of substance use type on COVID-19 Treatment Outcome

Nearly equal percentages of patients in each group required ICU admission (METH 21.9% vs. cannabis 15.2% vs. alcohol 20.5%, *P* = 0.7) and the mean length of stay in the ICU was non-significantly (*P* = 0.3) longer for cannabis users (6.9 ± 11.3 days) compared to METH (3.9 ± 6.7 days) and to alcohol users (2.0 ± 1.7 day). However, cannabis users had a significantly longer interval between a positive SARS-Cov-2 test and discharge from hospital (cannabis vs METH vs alcohol: 9.4 ± 9.2 vs 5.9 ± 6.3 vs 4.7 ± 4.7 days respectively, *P* = 0.01). More patients in the METH group compared to cannabis and alcohol groups required intubation, but the difference was not statistically significant (METH 15.6% vs. cannabis 8.7% vs. alcohol 9.1%, *P* = 0.5). About one third of all patients exhibited manifestations of delirium with no significant difference between the groups (METH 37.5% vs. cannabis 23.9% vs. alcohol 29.5%, *P* = 0.4). Compared to METH and cannabis users, more patients in the alcohol use group died within 10 months of SARS-CoV-2 positive test, but the difference was not significant (Alcohol vs METH vs cannabis: 9.1% vs 6.3% vs 4.3% respectively, *P* = 0.6) (Table 4).

**Table 4:** Association between outcomes and substance use group

	Group				
	METH (*N* = 32) n (%)	Cannabis (*N* = 46) n (%)	Alcohol (*N* = 44) n (%)	Total (*N* = 122) n (%)	*P*-value
**ICU LOS (in days)**					0.3471^1^
N (Missing)	7 (25)	7 (39)	9 (35)	23 (99)	
Mean (SD)	3.9 (6.69)	6.9 (11.28)	2.0 (1.73)	4.0 (7.23)	
Median	1	2	1	1	
IQR	1.0, 2.0	1.0, 7.0	1.0, 3.0	1.0, 3.0	
Range	1.0, 19.0	1.0, 32.0	1.0, 6.0	1.0, 32.0	
**Interval between positive COVID and discharge (in days)**					0.0167^1^
N (Not applicable)	23 (9)	27 (19)	44 (0)	94 (28)	
Mean (SD)	5.9 (6.34)	9.4 (9.20)	4.7 (4.65)	6.3 (6.87)	
Median	4	7	3	4	
IQR	2.0, 6.0	3.0, 13.0	1.0, 6.0	2.0, 7.0	
Range	1.0, 22.0	1.0, 40.0	1.0, 19.0	1.0, 40.0	
Admitted to ICU	7 (21.9%)	7 (15.2%)	9 (20.5%)	23 (18.9%)	0.7181^2^
Intubation Required	5 (15.6%)	4 (8.7%)	4 (9.1%)	13 (10.7%)	0.5687^2^
Delirium	12 (37.5%)	11 (23.9%)	13 (29.5%)	36 (29.5%)	0.4328^2^
Deceased	2 (6.3%)	2 (4.3%)	4 (9.1%)	8 (6.6%)	0.6596^2^

*ICU* Intensive Care Unit, SD Standard deviation, *IQR* Inter-quartile range

^1^Kruskal-Wallis *p*-value; ^2^Chi-Square *p*-value

Multivariate models revealed no statistically significant differences in ICU length of stay, interval between SARS CoV-2 positive test and discharge, ICU admission, mortality, delirium, and intubation between any comparison of METH, cannabis, and alcohol user cohorts after adjusting for covariates (Table 5). METH users had a non-significant trend (*P* = 0.068) towards increased risk for delirium compared to alcohol users. Cannabis users on the other hand had an increased risk of delirium as compared to METH users (OR: 2.14, *p*- value = 0.079). Cannabis users also had a longer interval between a positive test and discharge (RC: 0.45, *p*- value = 0.079), as compared to METH users, however not statistically significant.

**Table 5:** Adjusted association between the outcomes of interest and selected covariates

	**Alcohol vs METH (reference)**		**Cannabis vs METH (reference)**	
	OR (95% CI)	*P* value	OR (95% CI)	*P* value
ICU LOS: RC (95% CI)	-0.69 (-3.15, 1.77)	0.55	-0.2 (-2.24, 1.83)	0.83
Interval between SARS-CoV-2 positive test and discharge: RC (95% CI)	-0.35 (-0.88, 0.18)	0.20	0.45 (-0.05, 0.94)	0.079
ICU admission	1.62 (0.21, 12.28)	0.69	1.3 (0.28, 6.11)	0.98
Mortality	1.26 (0.15, 10.64)	0.69	0.72 (0.11, 4.8)	0.61
Delirium	0.32 (0.05, 2.14)	0.068	2.14 (0.38, 11.98)	0.079
Intubation	0.26 (0.02, 2.75)	0.21	0.93 (0.16, 5.49)	0.44

Adjusted for: Age, Sex, Vaccination before hospital admission, tobacco use, psych symptoms AMS, alcohol related disorders, stimulant usage, benzodiazepines s given

*RC* Regression coefficient, *OR* Odds ratio, *CI* Confidence interval, *ICU* Intensive care unit

## Discussion

The results of this study revealed strikingly similar high rates of medical and psychiatric comorbidities, as well as similar rates of severe COVID-19 illness as evident by ICU admission, in-hospital delirium, and post-hospitalization mortality rates in current METH, cannabis, and alcohol users. People who use psychoactive substances in general are less likely to have reliable access to care and often present with more advanced illnesses (Hossain et al. 2020). Therefore, our study provides a realistic snapshot of these patients compared to large-scale epidemiological studies that rely on health insurance or other national data sets. Equally important, our results suggest that individuals who currently use METH, cannabis, and alcohol have similar mortality rates during the first year following COVID-19 treatment. This specific finding is particularly concerning, since many patients in the cohort received their follow-up care at other institutions, so our post-discharge mortality rates may well be an underestimate.

Of note, significantly more individuals using alcohol received vaccination before their COVID-19 associated hospital visit compared to individuals who use METH or cannabis. There was no difference in vaccination rates following hospitalization. A potential cause of this discrepancy is that not all patients from all groups were admitted to the hospital at the same time. More of the individuals with high blood alcohol concentrations were evaluated in late 2021 and 2022 compared with individuals who use METH or cannabis. Therefore, there may be a timing bias in the groups’ likelihood to be vaccinated. Several studies have speculated about trends in COVID-19 vaccination in individuals with substance use disorders, but research is limited (Kumar et al. 2022). Concerns regarding COVID-19 vaccination in populations with substance use disorders include structural barriers, lack of trust in the health care system, lack of a strong patient-provider relationship, and racial disparities in the healthcare system (Kumar et al. 2022) (Barocas 2021; Mellis et al. 2021). The small sample size makes interpreting this observation challenging. One speculation could potentially be related to infection with different COVID-19 variants. However, further research is needed to probe this possibility.

The demographics of our patients show the expected male predominance in METH and alcohol users (Becker and Hu 2008) and the narrowing sex gap in cannabis users (Nia et al. 2018). The finding that only 12.5% of METH users, 30.4% of cannabis users, and 31.8% of alcohol users are married while 31.3%, 47.8%, and 20.5% respectively are employed suggests that these individuals are lacking marital support and the socioeconomic advantages of employment. High rates of medical and psychiatric comorbidities may contribute to the inability of these patients to maintain steady employment (Sherba et al. 2018). We saw lower rates of obesity (BMI > 30 kg/m^2^) in METH (19.4%) users compared to cannabis users (39.1%), which may be related to the appetite suppressant effects of stimulants (Rasmussen 2015). While cannabis is used to improve appetite (Grotenhermen 2003), the data about obesity among individuals who use cannabis is mixed (Fearby et al. 2022). Obesity in both populations could be attributed to comorbid medical conditions such as diabetes or hypothyroidism (Gierach et al. 2014) or to concomitant heavy alcohol drinking (Traversy and Chaput 2015). The prevalence of diabetes and hypothyroidism in our cohort is 21.7% in the cohort using cannabis and 34.4% in the cohort using METH which is higher than the national averages (Glovaci et al. 2019). Cannabis use is associated with higher rates of hypothyroidism and diabetes (Borowska et al. 2018). We have recently shown that cannabis administration modulates blood concentrations of insulin and other appetitive and metabolic hormones (Farokhnia et al. 2020).

Concomitant tobacco use was highly prevalent in our patients; 78.1%, 80.4%, and 75.0% of METH, cannabis, and alcohol users respectively reported current or past tobacco use. Other studies have found similarly high correlation between tobacco smoking and cannabis (Benowitz et al. 2019; Schauer et al. 2017) and also METH use (Sung et al. 2015; Yoon et al. 2021). Tobacco smoking has well known adverse pulmonary effects, and therefore can contribute to negative respiratory outcome of COVID-19 in these patient populations (Carrico et al. 2018).

The high rates of comorbid medical and psychiatric conditions in the three groups indicate some characteristic patterns (Hong et al. 2021; Connor et al. 2021; Lowe et al. 2019; Lee et al. 2022; Ciccarone and Shoptaw 2022). Most notably, their complex medical conditions were consistent with those affecting young individuals. Over half of the METH and cannabis users were younger than 40 years of age and all METH users were below 60 years old. The mean age of alcohol users was 44.8 as well, so these patients were also largely young. In addition, nicotine use is associated with high rates of medical comorbidities, and the majority of these cohorts were smokers (Tsai et al. 2020). Moreover, depression and anxiety typically affect young people (Racine et al. 2021) and are also associated with obesity (Fulton et al. 2022), early-onset diabetes (Roy and Lloyd 2012) and other medical conditions (DeJean et al. 2013). METH use, specifically through the intravenous route of administration, is associated with increased risk for hepatitis C, and patients with chronic pain conditions sometimes use cannabis as an analgesic (Romero-Sandoval et al. 2018). Over 50% of our patients had a documented history of comorbid mood disorder as well.

The use of COVID specific medications (Remdesivir, steroids, convalescent plasma, or monoclonal antibodies) was required in 25.0% of METH users, 26.1% of cannabis users, and 25.0% of alcohol users. The individuals in the alcohol use cohort were identified while hospitalized, so the hospitalization rate for the alcohol use group is 100%. However, the hospitalization rates of the METH users and cannabis users were also high at 71.9% and 58.7% respectively. Cannabis users had the highest interval between positive COVID-19 test and hospital discharge with a mean of 9.4 days compared to 5.9 days in METH users and 4.7 days in alcohol users (*P *= 0.017). Of note, this increased interval between positive COVID-19 test and hospital discharge did not remain significant when multivariate analysis was performed. 21.9% of METH users, 15.2% of cannabis users, and 20.5% of alcohol users required ICU level care during their admission which is higher than we had initially anticipated.

This high severity of illness is consistent with previous studies (Baillargeon et al. 2021; Benzano et al. 2021; Catalan et al. 2022; Wang et al. 2021; Parohan et al. 2020; Wolff et al. 2021; Zhou et al. 2021) and could explain, to some extent, the high rates of delirium, occurring in 37.5% of patients using METH, 23.9% of patients using cannabis, and 29.5% of patients using alcohol. We were also surprised to find the high rates of delirium across groups and that there were not significantly more alcohol users that experienced withdrawal delirium compared to cannabis and METH users. Substance use in general is associated with higher risk for the development of delirium (D'Orazio et al. 2010) and several of the alcohol users experienced withdrawal delirium; however, data regarding similar association with cannabis is scant (Gerlach et al. 2019). Moreover, the role of nicotine withdrawal in the delirium cannot be ruled out (Mayer et al. 2001). We have recently shown that acute nicotine withdrawal causes disruption in anterior cingulate glutamatergic homeostasis and functional connectivity (Abulseoud et al. 2020), and both factors contribute to delirium pathology (Wilson et al. 2020; Oh et al. 2019). Similarly, the possibility of alcohol withdrawal in the non-alcohol cohorts cannot be excluded, since we do not have data on alcohol consumption prior to presentation. In-hospital delirium is a known risk factor for higher mortality rates after discharge (Thomason and Ely 2004).

Eight patients total died within 10 months of positive SARS-CoV-2 PCR test. Two patients from the METH group (6.3%), two patients from the cannabis group (4.3%), and four patients from the alcohol group (9.1%) were deceased. This high mortality rate is surprisingly less than the 9.5% mortality reported by Wang et al. in their large-scale study (Wang et al. 2021).

Our results are different from a recent report by Shover et al. (Shover et al. 2022) They compared COVID-19 outcomes between current cannabis users (*n* = 69) and non-users (*n* = 1762) and found significant improvement in COVID-19 outcome among current cannabis users associated with decreased illness severity. Several factors could contribute to the difference between the Shover et al. study and the current results. First, we compared individuals currently using cannabis to those using METH or alcohol and not to non-users, because cannabis use is common among the general public. Therefore, we are not able to determine whether substance use influences COVID-19 hospital outcomes, but rather we are able to compare outcomes among individuals abusing different substances. Identifying a cannabis non-user based on negative urine drug screen or by not finding mention of cannabis use in the EMR is inaccurate. Second, only 20% of the individuals using cannabis in Shover et al.’s cohort reported current nicotine use compared to 80.4% in our subjects. Similarly, the cohort using cannabis in Shover et al.’s paper reported lower prevalence of comorbid medical conditions compared to our patients. For example, they found cardiovascular disease in 16% vs. 54.3% in our cohort, and kidney disease in 17% vs. 34.8% in our subjects. Third, they found no significant difference in survival. In our cohort, none of the patients using cannabis died during hospitalization, though two (out of 46) died within 1 year of discharge.

The study outcomes may have been affected by several limitations. These include the methodology of its retrospective design and relatively small sample size. In addition, the data collection did not include the extent of alcohol use, which may have been a confounding factor, particularly in patients developing in-hospital delirium. Because a urine drug test for cannabis can remain positive for weeks after use, there is no way to precisely identify when patients used cannabis (Thomason and Ely 2004). Additionally, as the study compared individuals using METH, cannabis, and alcohol, there was no COVID-19 negative control group. Because we collected self-reported tobacco use data amongst patients who were current and former users of tobacco, we were unable to separate current and past usage which may have influenced results.

Due to the small sample size, we were unable to include all potential variables including specific psychiatric and medical comorbidities in the multivariate analysis. Substance use was not quantified and relied on either problem lists or patient self-report which constitutes an additional limitation in the present study. Finally, the high degree of tobacco use may have been a confounding factor in risk of medical co-morbidities.

## Conclusion

In conclusion, individuals currently using cannabis or METH in this study had high rates of medical and psychiatric comorbidities and comparable courses of COVID-19 illness and mortality to each other as well as to individuals using alcohol. Individuals using cannabis had a significantly longer interval between SARS-CoV-2 positive test and hospital discharge, while significantly more individuals using alcohol were vaccinated for COVID-19 prior to hospitalization compared to the cohorts using METH and cannabis. Further research is needed to replicate these findings and explore rates and causes of post-COVID mortality among patients with substance use disorder in general and patients using cannabis specifically.

## Supplementary Information

**Additional file 1: Supplementary Table 1.** Comorbid medical and psychiatric conditions.

**Additional file 2: Supplementary Table 2.** Hospital clinical outcomes among study patients.

## Data Availability

Data is available upon request via email to the corresponding author, Osama Abulseoud, MD: Abulseoud.Osama@mayo.edu.
