# Supplementary material for: No difference in COVID-19 treatment outcomes among current methamphetamine, cannabis and alcohol users
Source: J Cannabis Res. 2023 Jun 19;5:23. doi: 10.1186/s42238-023-00193-w (PMC10280862; doi:10.1186/s42238-023-00193-w)
Supplement: Supplementary file 1 — Additional file 1: Supplementary Table 1. Comorbid medical and psychiatric conditions. [file 42238_2023_193_MOESM1_ESM.docx]

**Supplementary Table 1: Comorbid medical and psychiatric conditions**

|  | Study Group | | |  |  |
| --- | --- | --- | --- | --- | --- |
|  | METH (N=32)  n (%) | Cannabis (N=46)  n (%) | Alcohol (N=44)  n (%) | Total (N=122)  n (%) | P-value^1^ |
| Pulmonary conditions | 13 (40.6%) | 20 (43.5%) | 14 (31.8%) | 47 (38.5%) | 0.5 |
| Cardiovascular disorders | 14 (43.8%) | 25 (54.3%) | 28 (63.6%) | 67 (54.9%) | 0.2 |
| Renal/Male GU disorders | 14 (43.8%) | 16 (34.8%) | 18 (40.9%) | 48 (39.3%) | 0.7 |
| Gastrointestinal Issues | 13 (40.6%) | 14 (30.4%) | 19 (43.2%) | 46 (37.7%) | 0.4 |
| Endocrine disorders | 11 (34.4%) | 10 (21.7%) | 8 (18.2%) | 29 (23.8%) | 0.2 |
| Neurological conditions | 6 (18.8%) | 11 (23.9%) | 16 (36.4%) | 33 (27.0%) | 0.1 |
| Infection related conditions | 7 (21.9%) | 3 (6.5%) | 3 (6.8%) | 13 (10.7%) | 0.056 |
| Orthopedic conditions | 6 (18.8%) | 8 (17.4%) | 7 (15.9%) | 21 (17.2%) | 0.9 |
| Pain disorders | 6 (18.8%) | 10 (21.7%) | 10 (22.7%) | 26 (21.3%) | 0.9 |
| Cancer | 5 (15.6%) | 4 (8.7%) | 2 (4.5%) | 11 (9.0%) | 0.1 |
| Pregnancy/GYN conditions | 2 (6.3%) | 7 (15.2%) | 4 (9.1%) | 13 (10.7%) | 0.4 |
| Dermatologic conditions | 2 (6.3%) | 4 (8.7%) | 6 (13.6%) | 12 (9.8%) | 0.5 |
| ENT disorders | 1 (3.1%) | 0 (0.0%) | 3 (6.8%) | 4 (3.3%) | 0.1 |
| Others | 4 (12.5%) | 10 (21.7%) | 11 (25.0%) | 25 (20.5%) | <.0001 |
| Mood disorders | 18 (56.3%) | 27 (58.7%) | 24 (54.5%) | 69 (56.6%) | 0.9 |
| Anxiety disorders | 10 (31.3%) | 19 (41.3%) | 13 (29.5%) | 42 (34.4%) | 0.4 |
| Psychotic disorders | 4 (12.5%) | 6 (13.0%) | 14 (31.8%) | 24 (19.7%) | 0.04 |
| Suicide or self-harm | 3 (9.4%) | 1 (2.2%) | 7 (15.9%) | 11 (9.0%) | 0.07 |
| Alcohol use | 9 (28.1%) | 7 (15.2%) | 41 (93.2%) | 57 (46.7%) | <.0001 |
| Stimulant use | 20 (62.5%) | 8 (17.4%) | 5 (11.4%) | 33 (27.0%) | <.0001 |
| Sedative/ hypnotic use | 7 (21.9%) | 2 (4.3%) | 5 (11.4%) | 14 (11.5%) | 0.057 |
| Personality disorders | 0 (0.0%) | 5 (10.9%) | 3 (6.8%) | 8  (6.6%) | 0.1 |
| Delirium during previous hospitalization | 12 (37.5%) | 11 (23.9%) | 13 (29.5%) | 36 (29.5%) | 0.4 |
| Other psychiatric disorders | 6 (18.8%) | 11 (23.9%) | 14 (31.8%) | 31 (25.4%) | 0.4 |

^1^Chi-Square p-value.

GU = Genitourinary; GYN = Gynecologic; ENT = Ear, Nose and Throat
