# Supplementary material for: No difference in COVID-19 treatment outcomes among current methamphetamine, cannabis and alcohol users
Source: J Cannabis Res. 2023 Jun 19;5:23. doi: 10.1186/s42238-023-00193-w (PMC10280862; doi:10.1186/s42238-023-00193-w)
Supplement: Supplementary file 2 — Additional file 2: Supplementary Table 2. Hospital clinical outcomes among study patients. [file 42238_2023_193_MOESM2_ESM.docx]

**Supplementary Table 2: Hospital clinical outcomes among study patients**

|  | Group | | | |  |
| --- | --- | --- | --- | --- | --- |
|  | METH (N=32)  n (%) | Cannabis (N=46)  n (%) | Alcohol (N=44)  n (%) | Total (N=122)  n (%) | P-value |
| **Interval between vaccination and hospital visit (in days)** |  |  |  |  | 0.0799^1^ |
| N (Missing) | 2 (30) | 4 (42) | 17 (27) | 23 (99) |  |
| Mean (SD) | 31.0 (7.07) | 143.3 (31.06) | 221.2 (132.20) | 191.1 (127.64) |  |
| Median | 31 | 137.5 | 223 | 171 |  |
| IQR | 26.0, 36.0 | 119.5, 167.0 | 108.0, 340.0 | 89.0, 315.0 |  |
| Range | 26.0, 36.0 | 114.0, 184.0 | 19.0, 423.0 | 19.0, 423.0 |  |
| **Hospital admission** | 23 (71.9%) | 27 (58.7%) | 44 (100%) | 94 (77.0%) | <.0001^2^ |
| **COVID medications** | 8 (25.0%) | 12 (26.1%) | 11 (25.0%) | 31 (25.4%) | 0.9911^2^ |
| Remdesivir | 6 (18.8%) | 8 (17.4%) | 7 (15.9%) | 21 (17.2%) | 0.9481^2^ |
| Steroids | 6 (18.8%) | 9 (19.6%) | 8 (18.2%) | 23 (18.9%) | 0.9859^2^ |
| Convalescent plasma or monoclonal antibodies | 4 (12.5%) | 4 (8.7%) | 0 (0.0%) | 8 (6.6%) | 0.0715^2^ |
| **Opiate Pain Medications** | 7 (21.9%) | 16 (34.8%) | 11 (25.0%) | 34 (27.9%) | 0.3974^2^ |
| **Benzodiazepines** | 15 (46.9%) | 12 (26.1%) | 30 (68.2%) | 57 (46.7%) | 0.0003^2^ |
| **Developed delirium during this hospitalization** | 12 (37.5%) | 11 (23.9%) | 13 (29.5%) | 36 (29.5%) | 0.4328^2^ |
| **Received antipsychotics** | 7 (21.9%) | 13 (28.3%) | 9 (20.5%) | 29 (23.8%) | 0.6563^2^ |
| **Interval between SARS-CoV-2 positive test and death (in days)** |  |  |  |  | 0.6735^1^ |
| N (Not Applicable) | 2 (30) | 2 (44) | 4 (40) | 8 (114) |  |
| Mean (SD) | 131.5 (156.27) | 208.0 (120.21) | 237.3 (108.17) | 203.5 (112.72) |  |
| Median | 131.5 | 208 | 210.5 | 210.5 |  |
| IQR | 21.0, 242.0 | 123.0, 293.0 | 160.0, 314.5 | 132.0, 267.5 |  |
| Range | 21.0, 242.0 | 123.0, 293.0 | 141.0, 387.0 | 21.0, 387.0 |  |
| ^1^Kruskal-Wallis p-value; ^2^Chi-Square p-value. |  |  |  |  |  |

SD = standard deviation; IQR = Inter quartile range; ICU = Intensive Care Unit; LOS = Length of Stay.
